# Patients’ perceived purpose of clinical informed consent: Mill’s individual autonomy model is preferred

**DOI:** 10.1186/1472-6939-15-2

**Published:** 2014-01-10

**Authors:** Muhammad M Hammami, Eman A Al-Gaai, Yussuf Al-Jawarneh, Hala Amer, Muhammad B Hammami, Abdullah Eissa, Mohammad Al Qadire

**Affiliations:** 1 Clinical Studies and Empirical Ethics Department, King Faisal Specialist Hospital and Research Centre, P O Box # 3354 (MBC 03), Riyadh 11211, Saudi Arabia; 2 College of Medicine, Alfaisal University, Riyadh, Saudi Arabia

**Keywords:** Informed consent, Middle East, Norm perception, Current practice, Gender difference, Autonomy

## Abstract

**Background:**

Although informed consent is an integral part of clinical practice, its current doctrine remains mostly a matter of law and mainstream ethics rather than empirical research. There are scarce empirical data on patients’ perceived purpose of informed consent, which may include administrative routine/courtesy gesture, simple honest permission, informed permission, patient-clinician shared decision-making, and enabling patient’s self decision-making. Different purposes require different processes.

**Methods:**

We surveyed 488 adults who were planning to undergo or had recently undergone written informed consent-requiring procedures. Perceptions of informed consent purpose (from norm and current practice perspectives) were explored by asking respondents to rank (1 = most reflective) 10 randomly-presented statements: “meaningless routine”, “courtesy gesture” “litigation protection”, “take away compensation rights”, “inform patient’, “make sure patient understand”, “document patient’s decision”, “discover patient’s preferences”, “have shared decision”, and “help patient decide”.

**Results:**

Respondents’ mean (SD) age was 38.3 (12.5); 50.4% were males, 56.8% had ≥ college education, and 37.3% had undergone a procedure. From the norm perspective, the least reflective statement was “meaningless routine” (ranked 1–3 by 2.6% of respondents) and the most reflective statements were “help patient decide”, “make sure patient understand”, and “inform patient” (ranked 1–3 by 65%, 60%, and 48% of respondents with median [25%,75%] ranking scores of 2 [1,5], 3 [2,4], and 4 [2,5], respectively). Compared to their counterparts, males and pre-procedure respondents ranked “help patient decide” better, whereas females and post-procedure respondents ranked “inform patient” better (p = 0.007 to p < 0.001). Age was associated with better ranking of “help patient decide” and “make sure patient understand” statements (p < 0.001 and p = 0.002, respectively), which were ranked 1–3 by only 46% and 42% of respondents from the current practice perspective (median ranking score 4 [2,6], p < 0.001 vs. norm perspective for both).

**Conclusions:**

1) the informed consent process is important to patients, however, patients vary in their views of its purpose with the dominant view being enabling patients’ self decision-making, 2) males, pre-procedure, and older patients more favor a self decision-making purpose, whereas females and post-procedure patients more favor an information disclosure purpose, and 3) more self decision-making and more effective information disclosure than is currently practiced are desired. An informed consent process consistent with Mill’s individual autonomy model may be suitable for most patients.

## Background

Although the informed consent process has long been an integral part of clinical practice [1], its exact ethical purpose(s) remains controversial [2,3]. In addition to being a means for providing efficient health care [4] and building trust, the informed consent process has been conceptualized to have the moral goals of obtaining permission, supporting shared decision-making, and enabling autonomous decision-making [3]. In the 1970s, the informed consent was embraced as a correction to paternalism, however, in the 1980s and 1990s, shared decision-making was viewed as a necessary correction of “exaggerated individualism” [5].

The doctrine of informed consent has been imposed on medicine through nonmedical authorities [6] and continues to be primarily a legal and ethical concept rather than evidence-based [3]. In addition, the development of informed consent for clinical care purposes (clinical informed consent) has been affected by clinical research atrocities and the resulting regulations [7], despite important differences between clinical care and clinical research. In clinical care, patients seek care (informed request rather than informed consent) [8], benefits and risks are better defined, the aim of the interaction is to benefit the individual patient rather than patients in general [9], and clinical care practitioners are more formally trained and licensed. In the West, informed consent for surgery arose in the early 20th century as courts moved to protect patients from battery and negligence; the modern legal precedent for “simple” consent was written in 1914, whereas “informed” consent was first articulated in 1957 [3,6,10]. Interestingly, a forerunner of informed consent was documented in the Eastern Mediterranean region at least since the mid-17th century in the Registers of the Islamic Court of Candia (Heraklion) in Crete [11] and Tripoli-Syria [12]. The current medical practice law in Saudi Arabia is not dissimilar to the corresponding Western laws. The Saudi Arabian Ministry of Health Rules of Implementation for Regulation of the Practice of Medicine and Dentistry (1988) state that “prior to delivering medical treatment or carrying out an operative procedure, the legally competent patient’s consent, be he/she male or female, shall be obtained.”, and “the physician shall provide adequate explanation to the patient or his guardian on the nature of the medical treatment or operative procedure he intends to apply.” However, the Rules do not clarify what “adequate explanation” means nor address the issue of decision-making.

Philosophically, the informed consent is founded on the principle of Respect for Persons, which includes not only respect to autonomy but also to liberty and well-being. There are different accounts for autonomy, including Kantian, procedural, and Millian (also called perfectionist or substantive individual autonomy) accounts. Concentrating on one aspect of Respect for Persons principle or one interpretation of autonomy may be a cultural artifact [13,14]. The purpose of the informed consent process can be envisioned as a spectrum ranging from routine paper work/courtesy gesture, to obtaining honest permission (simple consent), to obtaining informed permission (informed consent), to reaching shared patient-clinician decision-making, to enabling patient’s self decision-making. Some patients and clinicians view the informed consent process as a ritualistic, bureaucratic compliance with social requirements that only substitute bureaucratic authority for professional authority [15-17]. According to Kantian account of autonomy, informed consent means obtaining honest permission by avoiding deception and coercion, without necessarily promoting personal deliberation and decision-making [18]. Supporters maintain that the exercise of informed personal deliberation is not an absolute right and does not have enough intrinsic value to justify a public policy or to have priority over other ethical considerations such as beneficence, justice, common good, and trust. According to the procedural account of autonomy [18,19], it is possible for an autonomous agent to have reflective but unconditional obedience, and thus the purpose of the informed consent process is to obtain an informed permission but not necessarily to promote participation in decision-making. The purpose is to afford patients the opportunity to be autonomous rather than forcing them to be so. In this regard, it has been argued that there are two types of information, information that is important in order to give permission, which should be understood, and information that is important in order to make an informed choice, which should be provided in an understandable way (but doesn’t necessarily need to be understood) [20]. Whereas only the first type of information would need to be disclosed according to Kantian account of autonomy, both types would need to be disclosed according to the procedural account of autonomy. In the space between procedural and substantive accounts of autonomy, the informed consent process can be viewed as a way to reach a shared patient-clinician decision-making [5]. This view places more emphasis on the responsibility of clinicians to promote both the well-being and autonomy of patients. It has been noted that the word “consent” derives from the Latin *con sentire,* which means to think or feel together [19,21]. Shared decision-making can take several forms. The clinician and patient could contribute information and a unique set of values and preferences, respectively, and then together agree on a course of treatment [22-24]. Alternatively, having agreed on the goals with the patient, the clinician can be free to make decisions on the best technical means to achieve them [3,25]. Support for the paradigm of shared decision-making has been recently articulated by an international consensus panel in the “Salzburg statement on shared decision-making [26]. According to Millian account of autonomy, it is important to promote patient’s self-reflection and enable patients to decide for themselves [18,19]. In contrast to the proceduralist account, which links autonomy with internal authenticity, the Millian account links autonomy to control, and autonomy is clearly differentiated from liberty (freedom of choice) and entails taking responsibility [10,19]. Patients can not simply trust the clinician to take good care of them; they can not freely decide to live in an obedient way, ignore information, or let others decide. They should control their course of treatment according to their point of view, a complex and dynamic outcome of not only judgments but also emotions, beliefs, desires, and habits. Although it has been suggested that autonomy as self-determination should be the governing principle of clinician-patient relationships [27], Millian individual autonomy may be problematic as a justification for public policy in a liberal democracy because it violates the neutrality principle and does not respect individuals who prefer not to decide for themselves or who do not have the richest autonomy resources [18]. Further, it has been argued that autonomy is only one of the characteristics of persons that require respect and that the moral purpose of informed consent should be primarily respect for persons, not promotion of autonomy [20].

Patients’ characteristics may affect their perceived purpose of the informed consent process. Individuals have different coping style, locus-of-control orientation, and health self-efficacy level (confidence level in effectively understanding the information, handling the task, and succeeding) [4], which may be in part, culture-dependent. For patients who cope by avoidance (rather than monitoring), information may be harmful; whereas patients with a strong internal locus-of-control orientation would welcome full Millian autonomy, patients with a strong external locus-of-control orientation (those who believe what happens to them is under the control of fate, chance, or powerful others) would be frightened if the decision-making weight is placed on their shoulders; and for patients with low health self-efficacy level, decision-making can be terrifying [4]. However, other things being equal, clinicians should aim to involve patients in decision making as much as possible because shared decision–making has been shown to improve care and reduce cost [28].

Since the informed consent doctrine is an integral part of clinical practice that strives to be evidence-based, it should be reshaped by empirical studies of patients’ perception [10,29]. There is a lack of studies to guide clinicians and policy makers on what patients like to see in the informed consent process, especially in Islamic/Arabic culture. The aim of this study was to explore how the purpose of the informed consent process is conceptualized by patients who are planning to undergo or who had recently undergone a written informed consent-requiring procedure in a tertiary care center in Saudi Arabia.

## Methods

The study employed a hybrid (theoretical and empirical) model to define patients’ perceived purpose(s) of the clinical informed consent process. Potential purposes of informed consent were identified through review of the literature. The empirical phase, a cross sectional survey of a convenience sample of tertiary care hospital attendees, was conducted in accordance with the ethical principles contained in the Declaration of Helsinki and after approval of the Research Ethics Committee (REC) of the King Faisal Specialist Hospital and Research Center (KFSH&RC). A request of waiver for written informed consent was approved by the REC and all respondents gave verbal consent.

Adult patients who had undergone a medical or surgical procedure requiring a specific written informed consent in the last 6 months or were planning to undergo one within the next 3 months, who were able to understand the purpose and procedures of the study, and who provided verbal informed consent were eligible to participate. The study was exploratory; sampling method and sample size were convenience-based with the aim to have around 500 evaluable responses. Participants were recruited by research coordinators in the waiting areas of the outpatients’ clinics. Research coordinators identified themselves as such to ensure that respondents would not give answers that they thought might be expected by healthcare professionals. The questionnaire was self-administered in Arabic language with research coordinators’ support as requested by respondents. A research coordinator was available at all times to assist respondents to complete the questionnaire and answered questions regarding the comprehension of the questionnaire. The following demographic data were collected, age, gender, whether the patient had undergone or was planning to undergo a procedure, and education level (illiterate, primary school, intermediate school, secondary school, college, university).

The questionnaire was developed by the authors in Arabic language based on literature review. During the development phase, we wanted to ensure that questionnaire’s statements will be understood by respondents as we have intended. This was iteratively evaluated by means of focused probing (cognitive-based testing) in the interview session following ordering of the statements. For example, respondents were asked, “what do you consider a shared decision between patient and clinician?”, “when would you say that the patient is making his own rational decision?”, “what do you think is the difference between informing someone of something and making sure that she/he understands”. In general, respondents had little difficulties in answering the probing questions related to the following statements, “inform patient', “make sure patient understand”, “document patient’s decision”, “discover patient’s preferences”, “have shared decision”, and “help patient decide”. Some respondents had difficulty differentiating “meaningless routine” from “courtesy gesture”, and most respondents considered “litigation protection” and “take away compensation rights” the same. The last four statements were not revised because they were, in part, intended to be a check on the internal consistency of responses. In total 20 different respondents were interviewed, 10 during face validity assessment and 10 during pilot testing of the final version (for acceptability, comprehensibility, and 2–3 days stability). We had to reword few statements during the face validity assessment phase but none during the pilot testing phase. The results of the pilot testing phase were not included in this report. The final questionnaire consisted of two parts: one on perception of norm and one on perception of current practice at KFSH&RC. Each part presented participants with 10 statements that covered potential purposes of the informed consent process: to enable/promote individual autonomy (“help patient decide”), to have patient-clinician shared decision (“discover patient’s preferences”, “have shared decision”), to disclose information (“inform patient”, “make sure patient understand”), to obtain permission/administrative (“take away compensation rights”, “litigation protection”, “document patient’s decision”), and bureaucratic ritual (“courtesy gesture”, “meaningless routine”). An English translation (accuracy confirmed by back translation) of the questionnaire and the instructions given to participants are available in the Additional file 1. The statements in each part of the questionnaire were presented to respondents in a random order. Respondents were asked to rank the statements in each part from 1 (most reflective) to 10 (least reflective).

Data were verified by double entry and validity checks were undertaken. The number (percentage) of respondents who gave ranks 1–3, 4–7, or 8–10 were determined for each statement. The mean (SD) and median [25%, 75%] ranking scores for each statement was calculated. Wilcoxon Signed Ranks test was used to compare perceptions of norm and current practice for each statement. Mann–Whitney test was used to compare males to females and respondents who had undergone a procedure to respondents who were planning to undergo one. Kruskal-Wallis test and Jonckheere-Terpstra test were used to compare ranking scores among 3 educational subgroups (up to intermediate school, secondary school/college, and university). Correlation between age and scores of each statement and between statements’ scores was studied using Spearman’s test. A 2-tailed p value of <0.01 was considered significant. 2-tailed p values are reported. Analyses were conducted using SPSS for Windows software (release 17.0.0, 2008. SPSS Inc., Chicago, ILL, USA).

## Results

Evaluable questionnaires were returned by 488 respondents. Thirty questionnaires (6.1%) had some missing data and 6 (1.2%) gave the same rank to more than one statement. 96.9% of the respondents were Saudis, 2.5% Non-Saudi Arabs, and 0.6% of other nationalities. Other respondents’ characteristics are summarized in Table 1.

**Table 1:** Characteristics of study respondents (no. = 488)

**Age-mean (SD), yr**	38.3 (12.5)
**Gender-no. (%)**	
Male	246 (50.4)
Female	242 (49.6)
**Procedure/surgery-no. (%)**	
Had in previous 6 months	182 (37.3)
Will have within 3 months	306 (62.7)
**Education level-no. (%)**	
Illiterate	16 (3.3)
Primary school	29 (6.0)
Intermediate school	46 (9.5)
Secondary school	119 (24.5)
College	58 (11.9)
University	218 (44.9)

Two respondents did not indicate their education level.

### Perceived purpose of clinical informed consent, norm perspective

Respondents ranked 10 statements (Table 2 and Additional file 1) related to potential purposes of clinical informed consent from 1 (most reflective) to 10, according to their perception of norm. As shown in Table 2, mean and median scores ranged from 3.02 and 2 for “help patient decide” statement to 8.89 and 9 for “meaningless routine” statement. The coefficient of variation (SD/mean) ranged from 18% for “meaningless routine” statement to 71% for “help patient decide” statement. Further, five of the 10 statements were ranked 1–3 by more than 30% of respondents, indicating diversity in norm perception.

**Table 2:** Patients’ perceived function of clinical informed consent: norm vs. current practice

**Statement abbreviation**	**Norm**		**Current practice**		**p Value**
	**Mean (SD)**	**Median [25%, 75%]**	**Mean (SD)**	**Median [25%, 75%]**	
“Help patient decide”	3.02 (2.15)	2 [1,5]	4.02 (2.48)	4 [2,6]	<001
“Make sure patient understand”	3.27 (1.93)	3 [2,4]	4.18 (2.38)	4 [2,6]	<001
“Inform patient”	3.86 (2.05)	4 [2,5]	3.93 (2.17)	4 [2,5]	0.67
“Have shared decision”	4.19 (2.05)	4 [3,6]	4.33 (2.24)	4 [3,6]	0.37
“Discover patient’s preferences”	4.32 (1.87)	4 [3,6]	5.04 (2.12)	5 [3,6]	<001
“Document patient’s decision”	4.83 (2.18)	5 [3,6]	4.31 (2.40)	4 [2,6]	<001
“Litigation protection”	7.08 (2.15)	7 [6,9]	6.30 (2.99)	7 [4,9]	<001
“Courtesy gesture”	7.81 (2.29)	9 [7,9]	7.48 (2.45)	8 [6,9]	0.006
“Take away compensation rights”	7.71 (1.89)	8 [7,9]	6.81 (2.54)	8 [6,9]	<001
“Meaningless routine”	8.89 (1.63)	9 [8,10]	8.49 (2.15)	9 [8,10]	0.003

Data are ranking scores for each of ten randomly-presented statements representing potential purposes of clinical informed consent. Respondents ranked each statement from 1 (most reflective) to 10. The number of responses for norm and current practice ranged from 466 to 467 and from 478 to 482, respectively. P value is for Wilcoxon Signed Ranks test. For full description of the statements, see the text and Additional file 1.

**Figure 1:**
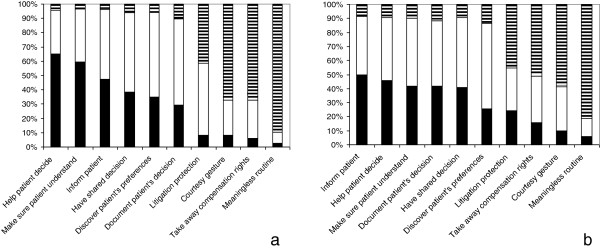
**Patients’ perception of the purpose of clinical informed consent (a, norm perception; b, current practice perception).** Data represent percentage of time each statement was ranked 1 to 3 (highest ranking, black bars), 4 to 7 (intermediate ranking, open bars), or 8 to 10 (lowest ranking, bars with horizontal lines).

The three statements with the best overall ranks, “help patient decide”, “make sure patient understand”, and “inform patient” were ranked 1–3 by 65%, 60%, and 48% of respondents, respectively. The three statements with the worse overall ranks, “meaningless routine”, “take away compensation rights”, and “courtesy gesture” were ranked 8–10 by 90%, 67%, and 67% of respondents, respectively. The data are presented in Figure 1. Only 2.6% and 8.1% of respondents, respectively, ranked “meaningless routine” and “courtesy gesture” statements 1–3, with an overall median rank of 9 for each statement (Table 2), suggesting that clinical informed consent is conceived to serve an important purpose by almost all respondents.

The differences between the ranking of “help patient decide” statement on one hand and “inform patient” and “have shared decision” statements on the other were statistically significant (p < 0.001), whereas the difference between the ranking of “help patient decide” statement and “make sure patient understand” statement was of borderline significance (p = 0.03). Further, there was significant negative correlation between ranking of “help patient decide” statement and three of the competing statements, “make sure patient understand” (rho = -0.14, p = 0.003), “inform patient” (rho = -0.2, p < 0.001), and “discover patient’s preferences” (rho = -0.15, p = 0.001), but not “have shared decision” statement (rho = -0.07, p = 0.15). The data indicate some degree of polarization in norm perceptions.

As shown in Table 3, there were significant differences between males and females in ranking “help patient decide” and “inform patient” statements. Further, “help patient decide” statement was ranked 1–3 by 71% of males and 59% of females, whereas “inform patient” statement was ranked 1–3 by 39% of males and 57% of females, suggesting that females are more likely to perceive the purpose of informed consent as information disclosure and males are more likely to perceive it as enabling self decision-making. Nevertheless, males and females when analyzed separately continued to rank “help patient decide” statement first followed by “make sure patient understand” and “inform patient” statements.

**Table 3:** Patients’ perceived purpose of clinical informed consent: males vs. females

	**Males**		**Females**		
**Statement abbreviation**	**Mean (SD)**	**Median [25%, 75%]**	**Mean (SD)**	**Median [25%, 75%]**	**p value**
**Norm**					
“Help patient decide”	2.76 (2.07)	2 [1,4]	3.29 (2.21)	3 [1,5]	0.007
“Make sure patient understand”	3.17 (2.06)	3 [2,4]	3.38 (1.78)	3 [2,4]	0.05
“Inform patient”	4.17 (2.00)	4 [3,5]	3.53 (2.06)	3 [2,5]	<001
“Have shared decision”	4.24 (2.06)	4 [3,6]	4.14 (2.04)	4 [3,5]	0.66
“Discover patient’s preferences”	4.47 (1.89)	4 [3,6]	4.17 (1.83)	4 [3,5]	0.06
“Document patient’s decision”	4.70 (2.12)	5 [3,6]	4.96 (2.24)	5 [3,6]	0.18
“Litigation protection”	6.95 (2.22)	7 [6,9]	7.23 (2.06)	7 [6,9]	0.23
“Courtesy gesture”	8.01 (2.08)	9 [7,9]	7.60 (2.48)	8 [7,9]	0.15
“Take away compensation rights”	7.58 (1.87)	8 [7,9]	7.85 (1.91)	8 [7,9]	0.03
“Meaningless routine”	8.93 (1.64)	9 [8,10]	8.85 (1.62)	9 [8,10]	0.23
**Current practice**					
“Help patient decide”	4.21 (2.57)	4 [2,6]	3.82 (2.37)	4 [1.8,6]	0.13
“Make sure patient understand”	4.30 (2.57)	4 [2,6]	4.06 (2.17)	4 [2,5]	0.61
“Inform patient”	4.29 (2.23)	4 [2.3,6]	3.55 (2.05)	3 [2,5]	<001
“Have shared decision”	4.58 (2.24)	4 [3,6]	4.08 (2.21)	4 [2,6]	0.009
“Discover patient’s preferences”	5.16 (2.08)	5 [4,7]	4.92 (2.15)	5 [3,6]	0.13
“Document patient’s decision”	4.16 (2.39)	4 [2,6]	4.47 (2.41)	4 [2,6]	0.13
“Litigation protection”	6.07 (3.15)	7 [2.3,9]	6.53 (2.81)	7 [5,8]	0.32
“Courtesy gesture”	7.44 (2.51)	8 [6,9]	7.53 (2.38)	8 [6,9]	0.15
“Take away compensation rights”	6.33 (2.70)	7 [4,8]	7.31 (2.26)	8 [7,9]	<001
“Meaningless routine”	8.36 (2.34)	9 [8,10]	8.63 (1.93)	9 [8,10]	0.77

Data are ranking scores for each of ten randomly-presented statements representing potential purposes of clinical informed consent. Respondents ranked each statement from 1 (most reflective) to 10. The number of responses for males and females ranged from 237 to 244 and from 229 to 238, respectively. P value is for Mann–Whitney test. For full description of the statements, see the text and Additional file 1.

As shown in Table 4, there were significant differences between pre- and post- procedure respondents in ranking the following statements, “help patient decide”, “inform patient”, and “have shared decision”. Further, “help patient decide” statement was ranked 1–3 by 70% of pre-procedure and 57% of post-procedure respondents, “inform patient” statement was ranked 1–3 by 41% of pre-procedure and 58% of post-procedure respondents, and “have shared decision” statement was ranked 1–3 by 44% of pre-procedure and 29% of post-procedure respondents. Furthermore, the difference in ranking of “make sure patient understand” statement between the two subgroups was of borderline significance (ranked 1–3 by 55% of pre-procedure and 68% of post-procedure respondents). Moreover, although pre-procedure respondents analyzed separately continued to rank “help patient decide” statement first and “make sure patient understand” statement second, they ranked “have shared decision” statement before “inform patient” statement. On the other hand, post-procedure respondents analyzed separately ranked “help patient decide” statement third (after “make sure patient understand” and “inform patient” statements). Together, the data suggest that having experienced an informed consent process/procedure may change norm perception of the purpose of informed consent process from involvement in decision-making toward information disclosure.

**Table 4:** Patients’ perceived purpose of clinical informed consent: pre- vs. post- procedure

	**Pre-procedure**		**Post-procedure**		
**Statement abbreviation**	**Mean (SD)**	**Median [25%,75%]**	**Mean (SD)**	**Median [25%,75%]**	**p value**
**Norm**					
“Help patient decide”	2.77 (2.06)	2 [1,4]	3.45 (2.24)	3 [1,5]	0.001
“Make sure patient understand”	3.42 (1.95)	3 [2,5]	3.02 (1.86)	3 [2,4]	0.02
“Inform patient”	4.14 (2.08)	4 [2,6]	3.38 (1.91)	3 [2,5]	<001
“Have shared decision”	3.96 (2.00)	4 [2,5]	4.58 (2.07)	5 [3,6]	0.001
“Discover patient’s preferences”	4.30 (1.84)	4 [3,5]	4.37 (1.91)	4 [3,6]	0.65
“Document patient’s decision”	4.71 (2.20)	5 [3,6]	5.03 (2.12)	5 [4,6]	0.13
“Litigation protection”	7.02 (2.09)	7 [6,9]	7.19 (2.23)	7 [6,9]	0.16
“Courtesy gesture”	7.49 (2.16)	9 [7,9]	7.58 (2.49)	9 [7,9]	0.26
“Take away compensation rights”	7.78 (1.78)	8 [7,9]	7.59 (2.06)	8 [7,9]	0.48
“Meaningless routine”	8.94 (1.57)	9 [8,10]	8.81 (1.72)	9 [8,10]	0.49
**Current practice**					
“Help patient decide”	3.88 (2.49)	4 [2,6]	4.25 (2.45)	4 [2,6]	0.08
“Make sure patient understand”	4.13 (2.27)	4 [2,6]	4.27 (2.56)	4 [2,6]	0.87
“Inform patient”	3.83 (2.05)	3 [2,5]	4.09 (2.36)	4 [2,6]	0.40
“Have shared decision”	4.19 (2.27)	4 [2,6]	4.59 (2.17)	4 [3,6]	0.03
“Discover patient’s preferences”	4.81 (2.11)	5 [3,6]	5.44 (2.07)	5 (4,7]	0.001
“Document patient’s decision”	4.38 (2.51)	4 [2,6]	4.20 (2.22)	4 [2,6]	0.57
“Litigation protection”	6.57 (2.74)	7 [5,9]	5.81 (3.34)	7 [2,9]	0.08
“Courtesy gesture”	7.54 (2.38)	8 [6,9]	7.39 (2.57)	9 [6,9]	0.74
“Take away compensation rights”	6.95 (2.49)	8 [6,9]	6.58 (2.63)	7 [4,8]	0.23
“Meaningless routine”	8.61 (1.98)	9 [8,10]	8.30 (2.42)	9 [8,10]	0.73

Data are ranking scores for each of ten randomly-presented statements representing potential purposes of clinical informed consent. Respondents ranked each statement from 1 (most reflective) to 10. The number of responses for pre- and post- procedure ranged from 291 to 305 and from 175 to 177, respectively. P value is for Mann–Whitney test. For full description of the statements, see the text and Additional file 1.

Age correlated negatively with ranking scores of “help patient decide” and “make sure patient understand” statements (rho = -0.19, p < 0.001; rho = -0.14, p = 0.002) and positively with ranking scores of “meaningless routine” statement (rho = 0.12, p = 0.008). Further, there was a positive correlation between age and ranking scores of “courtesy gesture” and ‘inform patient” statements of borderline significance (rho = 0.12, p = 0.01; rho = 0.12, p = 0.01, respectively). Together, the data suggest that older patients are more likely to attach importance to the informed consent process and to perceive its purpose as enabling self decision-making.

Because of small numbers, we combined respondents based on educational level into three groups, up to intermediate school education (n = 91), secondary school/college education (n = 177), and university education (n = 218). Educational level correlated only with the ranking score of “litigation protection” statement with mean (SD) and median [25%, 75%] scores of 6.66 (2.30) and 7 [6,8], 6.92 (2.17) and 7 [6,8], and 7.38 (2.03) and 7 [6.5,9], respectively (p = 0.01, and 0.003 for trend), suggesting that respondents with higher education level are less likely to perceive the purpose of the informed consent process as litigation protection.

### Perceived purpose of clinical informed consent, current practice perspective

Respondents also ranked the same 10 statements from 1 (most reflective) to 10 according to their perception of current practice. As shown in Table 2, mean and median scores ranged from 3.93 and 4 for “inform patient” statement to 8.49 and 9 for “meaningless routine” statement. The three statements with the best overall ranks, “inform patient”, “help patient decide”, and “make sure patient understand” were ranked 1–3 by 50%, 46%, and 42% of respondents, respectively. The three statements with worse overall rank, “meaningless routine”, “courtesy gesture” and “take away compensation rights” were ranked 8–10 by 81%, 59%, and 51% of respondents, respectively. The data are presented in Figure 1.

Ranking of “litigation protection” statement was positively correlated with ranking of “take away compensation rights” statement (rho = 0.45, p < 0.001), ranking of “have shared decision” statement was positively correlated with ranking of “help patient decide” (rho = 0.18, p < 0.001) and “discover patient’s preferences” statements (rho = 0.18, p < 0.001), and ranking of “inform patient” statement was positively correlated with ranking of “make sure patient understand” statement (rho = 0.10, p < 0.001), suggesting internal consistency of responses.

As shown in Table 3, there were significant differences between males and females in ranking the following statements, “inform patient”, “have shared decision”, and “take away compensation rights”. Further, “inform patient” statement was ranked 1–3 by 44% of males and 55% of females, “have shared decision” statement was ranked 1–3 by 34% of males and 48% of females, and “take away compensation rights” statement was ranked 1–3 by 22% of males and 9% of females. Moreover, when analyzed separately, males gave the best rank to “document patient’s decision” statement and females gave the best rank to “inform patient” statement (Table 3). Together, the data suggest that compared to males, females are more likely to perceive current practice as consistent with a patient-centered approach.

As shown in Table 4, there was significant difference between pre- and post- procedure respondents in ranking “discover patient’s preferences” statement. The difference in ranking “have shared decision” statement and “litigation protection” statement was of borderline significance. Further, “discover patient’s preferences” statement was ranked 1–3 by 29% of pre-procedure and 19% of post-procedure respondents, “have shared decision” statement was ranked 1–3 by 43% of pre-procedure and 37% of post-procedure respondents, and “litigation protection” statement was ranked 1–3 by 19% of pre-procedure and 34% of post-procedure respondents, suggesting that having experienced an informed consent process/procedure is associated with patients’ perception of current practice as less consistent with a patient-centered approach.

### Comparison of perceptions of norm and current practices

As shown in Table 2, ranking was significantly different between the two perspectives for all statements except “inform patient” and “have shared decision”. Non-patient-centered statements (“meaningless routine”, “take away compensation rights”, “courtesy gesture”, ‘litigation protection”, and “document patient’s decision”) were ranked better, whereas patient-centered statements (“help patient decide”, “make sure patient understand”, and “discover patient’s preferences”) were ranked worse according to perception of current practice. Further, there was a weak and insignificant correlation between the two perspectives for “help patient decide” statement (rho = 0.09, p = 0.06) and “have shared decision” statement (rho = 0.1, p = 0.04). Furthermore, age and ranking scores of “help patient decide” statement correlated negatively according to perception of norm (rho = -0.19, p < 0.001) and positively according to perception of current practice (rho = 0.08, p = 0.10). Moreover, the difference between the two perspectives in ranking score of “help patient decide” statement was larger in males than females (Table 3), suggesting the presence of some degree of patients’ dissatisfaction with the current informed consent process, especially in males and older patients.

## Discussion

The main aim of this study was to obtain empirical data on patients’ norm perception of the purpose of clinical informed consent in Saudi Arabia. Secondary aims were to explore whether norm perception is associated with certain demographics and how it compares to perception of current practice. The strengths of the study include, directly comparing various potential purposes, relatively large sample size, simultaneous examination of perceptions of norm and current practice, surveying actual patients rather than the general public, and uniquely addressing Arabic/Islamic culture. We found that: 1) the informed consent process is important to patients, however, patients vary in their views of its purpose with the dominant view being enabling patients’ self decision-making, 2) males, pre-procedure, and older patients more favor a self decision-making purpose, whereas females and post-procedure patients more favor an information disclosure purpose, and 3) more self decision-making and more effective information disclosure than is currently practiced are desired. The results suggest that an informed consent process based on Mill’s individual autonomy model may be suitable for most patients, especially male and older patients.

There are rather contradictory formations of the informed consent, including traditionalism, liability, and decision-making [30], with views ranging from a mere administrative document that a patient signs to an on-going communication process and decision-making. The informed consent is commonly claimed as the key to respecting patient’s autonomy; however, it has been argued that this claim is deeply obscure as there are many distinct conceptions of autonomy in circulation [8]. Further, since the informed consent process is integral to the practice of medicine, it should be evidence-based [10,29]. Moreover, clinical informed consent is important to patients [31-34], a fact that was confirmed by our data showing that only a small minority of respondents perceived it as a “meaningless routine” or a “courtesy gesture”.

### Diversity in norm perception of informed consent’s purpose

We found considerable diversity in norm perception of the informed consent’s purpose, indicated by large coefficients of variation of statements’ ranking scores, the fact that 5 statements were ranked 1–3 by more than 30% of respondents, and the fact that there were significant negative correlations between competing statements. The observed diversity suggests that a one-size-fits-all informed consent process may results in some degree of public dissatisfaction.

Understanding cultural expectations can provide insight into people perceptions. Arabic and Islamic societies are still influenced by Islamic social ethics. For example, the Saudi Arabian Ministry of Health Rules of Implementation for Regulation of the Practice of Medicine and Dentistry (1988) was based on the resolution rendered by the Committee of Senior Ulema (Arabic for religious scholars). Diversity in norm perception could be due to an absence of norm, that the norm is not well known, or that there are several rather than one norm. The later is more likely. Muslims come from several schools of thought and there is no statement in Al Quran or Prophet Muhammad’s Sayings that directly address the issue. Nevertheless, some generalizations can be made. 1) Al Quran encourages taking good care of one’s body as well as seeking treatment (Chapter 16, verses 68–69 and Chapter 2, verse 195) [35]. Prophet Muhammad said, "There is no disease that Allah has created, except that He also has created its remedy." (Sahih al-Bukhari 5678) [36] and “…your body has a right on you…” (Sunan Abudawud 1369) [37]. However, the issue may be more complicated. Based on the magnitude and certainty of benefits (and risks), seeking treatment could range from being a must, to permitted, to discouraged [38]. Further, for many health situations there may not be a clearly superior course of action [28], and choosing a course of action is determined by individual beliefs (cognitive) that an action leads to certain outcomes, and values (affective) regarding the outcomes. 2) Al Quran prohibits following others blindly without knowing their evidence (Chapter 33, verse 67; Chapter 43, verse 22; and Chapter 2, verse 111) [35]. 3) Al Quran encourages shared-decision making. It says, “And consult them in affairs.” (Chapter 3, verse 159), “..who (conduct) their affairs by mutual Consultation…” (Chapter 42, verse 38), and “If they both (mother and father) decide on weaning, by mutual consent, and after due consultation, there is no blame on them.” (Chapter 2, verse 233) [35]. 4) Finally, Al Quran forbids suspicion and undue distrust (Chapter 49, verse 12), permits delegation of decision-making (Chapter 18, verse 19), and emphasizes personal responsibility (Chapter 2, verse 286) [35]. Thus although Islam as a religion is centered on a divine law and obedience, the obedience is reflective and restricted (to Allah and the Prophet). Further, Islam does promote a sort of rational autonomy conceived as the ability to rationally determine what is in one’s best interest and as having the motivation to live accordingly [39]. It is of note that documentation of the use of formal informed consent for surgery in the Islamic/Arabic culture dates back at least to the 17th century [11,12].

### The dominant patients’ perception of informed consent’s purpose is enabling patients to make their own decisions

We found that “help patient decide” statement was assigned the best overall rank, was ranked 1–3 (out of 10) by 65% of respondents, and was ranked significantly different from the competing statements, indicating that the dominant patients’ view of informed consent’s purpose is enabling patients’ self decision-making. A New Zealand study with a different design showed that 64% of respondents preferred to take sole responsibility to decide which procedure to undergo, 31% preferred to be guided by the surgeon, and 5% preferred a brief explanation only [40]. In contrast, older data from North America showed that 57% of patients preferred to delegate their decisions to others [41,42]. Building the trust needed to allow patient to make the “leap of faith” to a surgeon’s care may be more important to some patients than participating in decision-making [16,17].

According to Millian individual autonomy, a slave does not gain autonomy by approving his chains, and a monk is not autonomous even if he autonomously chooses to abide by his superiors [19]. If patients are allowed to waive their interest in decision-making and decide to trust their clinicians, their freedom of choice is increased but their personal autonomy is decreased. It has been argued that although patients might long to throw themselves into the clinician’s caring arms, clinicians should not make decisions for patients since the consequences of the patient’s choice are not shared [43]. Choosing the stance of Millian individual autonomy rather than the stance of shared decision-making or delegation of decision-making may depend on factors related to the delegator, the decision [23], or the delegatee. Patients with a strong internal locus-of-control orientation, a monitoring style of coping, and a strong sense of health self-efficacy are most likely to prefer/benefit from a full respect for a full individual autonomy [4]. Factors related to the decision include, familiarity, ambiguity, significance, complexity, accountability, and time and money constrains. Of note, the 2003 US National Assessment of Adult Literacy by the National Center for Education Statistics showed that 36% of adults have basic or below basic health literacy (the degree to which individuals have the capacity to obtain, process, and understand basic health information and services needed to make appropriate health decisions) [44]. Factors related to the delegatee include trustworthiness, which depends on knowledge, ability, and motivation. The results of our study may be interpreted as showing a strong individual autonomy orientation, a strong sense of health self-efficacy, or reduced trust in the healthcare system.

### The perception of informed consent’s purpose is associated with gender and age

Although “help patient decide” statement continued to be ranked first when males and females were analyzed separately, its median ranking score was significantly more favorable in males compared to females. The opposite was true for “inform patient” statement, suggesting that females are more likely to perceive the purpose of the informed consent process as information disclosure and males are more likely to perceive it as enabling self decision-making. The results could be explained by the biological origin theory or the social role theory. Cross-cultural comparisons of gender differences would be important to differentiate between the two theories since gender role and stereotypes vary across cultures. It is not clear if our results are related to the observation that women’s greater perceived likelihood and severity of negative outcomes and lesser expectation of enjoyment partially mediate their lower propensity toward risky choices in healthcare and other domains [45]. The results do not necessarily indicate that female respondents in our study are less autonomous, as it is possible to forgo one’s autonomy in a specific relation and still retain it in general. Our female respondents could have found themselves less acquainted with healthcare decisions, could have more trust in the healthcare system, or could have been occupied with more important decisions. We have previously found gender differences in norm perception of consenting options for posthumous organ donation [46] but not of consenting for retrospective research [47].

We found significance association between age and ranking of statements favoring self decision-making. Older patients may assign more relative importance to healthcare decisions, or they may have higher perceived health self-efficacy (being more likely than younger respondents to be involved previously in healthcare decision-making), or both.

### The perception of informed consent’s purpose is associated with time in relation to procedure

We found that pre-procedure respondents ranked “help patient decide” statement first, whereas post-procedure respondents ranked “make sure patient understand” statement first. Further, the differences between the two subgroups in ranking “help patient decide”, “inform patient”, and “have shared decision” statements were significant, suggesting that having experienced and informed consent process/procedure may change norm perception of the purpose of informed consent from involvement in decision-making toward information disclosure. The perception of post-procedure respondents is consistent with Kantian account of autonomy and the principle of non-exploitation (rather than necessarily non-paternalism). According to this account, the purpose of the informed consent process is to provide assurance that patients are neither deceived nor coerced [8,18]. Autonomy in this view is synonymous with practical reason, where consent requires not only freedom from external influences and freedom from ignorance but also freedom from inner compulsions [5,18], which may be difficult for a patient to achieve. It is of note that doctors are advised against taking care of themselves as patients, to a major part, because of potential loss of objectivity [48-50]. The perception of post-procedure respondents is also consistent with procedural account of autonomy. A procedurally autonomous individual might on (periodic) reflection endorse an unconditionally obedient behavior [18], which may occur if there is high-level of trust. In this regard, it has been argued that the substitution of someone else’s judgment for the patient’s judgment about how to act may represent failure to respect the patient’s autonomy [18,20], that the informed consent process should be reconceptualized as a less “individualistic” and more “relational” [51], that patient’s autonomy should be seen as one among many values, including sympathy and patient’s well being [29], and that by moving from a physician-centered to a patient-centered decision- making one risks replacing a conservative “doctor-knows-best” paternalism with a new liberal paternalism, leading to a provider-patient relationships that are more impersonal and commercial [3,5]. The observed differences in perception of informed consent’s purpose in relation to time of procedure should be taken into account in designing future studies.

### More self decision-making and more understanding are desired compared to current practice

We found significant differences between norm and current practice perceptions of the purpose of informed consent. Non-patient-centered statements were ranked better, whereas patient-centered statements were ranked worse according to perception of current practice. Similar to our results, previous studies showed that 67% of respondents considered informed consent as a means of obtaining permission and only 18% agreed with its implications in terms of self-autonomy [40], that 46% and 68% of patients, respectively, believed that the main function of informed consent is to protect hospitals from litigation and to allow doctors to assume control [52], and that observance of medical ethics in term of obtaining adequate informed consent was inadequate [53]. A similar difference between perceptions of norm and current practice was found in the same population in relation to consenting options for posthumous organ donation [46] and consenting for retrospective research [47]. Together with our finding that age correlated with ranking scores of “help patient decide” statement in opposite directions according to the two perspectives, and that the difference between the two perspectives was more pronounced in males than females, the results suggest the presence of some degree of patients’ dissatisfaction with the current informed consent process, especially in males and older patients.

### Study limitations

Important considerations in the interpretation of the findings of the study include, that it was based on convenience sampling, that it was performed in a single tertiary healthcare institution in a major metropolitan city, and that the enrolment criteria resulted in selection of individuals with higher education. Thus the results may not be generalizable. However, it is of note that the institution is a governmental referral center for the entire country, that educational level was in general not associated with statements’ ranking scores, and that the enrollment criteria were chosen to achieve a balance between study’s internal validity and external validity. Another important consideration is the possibility that not all respondents understood the statements as we have intended or were able to differentiate between them. It is not likely that such bias would be large enough to alter the main conclusions of the study taking into consideration the results of cognitive-based testing during the developmental phase of the questionnaire, the enrollment criteria (patients who had or are planning to have a written consent-requiring procedure rather than members of the general public, patients who are able to understand the purpose and procedures of the study), study methodology (availability of a research coordinator to assist, respondents were forced to carefully consider each statement since the 10 statements were presented together and they had to rank them 1 to 10 using each number once), and the observed internal consistency of responses (indicated by predicted association in the ranking of certain statements). Further, the study only addressed written informed consent-requiring procedures and the results may not apply to other healthcare situations with lower risks and/or simpler decisions. Finally, it should be noted that since public opinion regarding the informed consent process would be expected to continue to evolve, the results may not be extrapolateable in time.

## Conclusions

This study contributes to an important but rather neglected area of healthcare research. The purpose of informed consent for clinical care is rooted in concepts that have been formed after a series of political and philosophical developments rather than empirical studies. Different informed consent purposes would require different processes. In the setting of outpatient clinics at a tertiary care hospital in Saudi Arabia, we found that: 1) the informed consent process is important to patients, however, patients vary in their views of its purpose with the dominant view being enabling patients’ self decision-making, 2) males, pre-procedure, and older patients more favor a self decision-making purpose, whereas females and post-procedure patients more favor an information disclosure purpose, and 3) more self decision-making and more effective information disclosure than is currently practiced are desired. The results suggest that Mill’s individual autonomy model of informed consent is preferred and that an informed consent process consistent with this model may be suitable for most patients, especially males and older patients. The results confirm that there may be some degree of patients’ dissatisfaction with the informed consent process in current practice. Finally, the observed differences in perception of informed consent’s purpose in relation to time of procedure should be taken into account in designing future studies.

## Competing interests

The authors declare that they have no competing interests.

## Authors’ contributions

MMH designed the study, performed statistical analysis, and wrote the manuscript. EAG, YJ, HA, and AE participated in data collection. MBH participated in statistical analysis and literature review and co-wrote the manuscript. MAQ participated in study design and literature review. All authors read and approved the final manuscript.

## Pre-publication history

The pre-publication history for this paper can be accessed here:

http://www.biomedcentral.com/1472-6939/15/2/prepub

## Supplementary Material

Additional file 1 Study Questionnaire: An English translation of the questionnaire and instructions given to participants. Click here for file
