# Supplementary material for: Patients’ perceived purpose of clinical informed consent: Mill’s individual autonomy model is preferred
Source: BMC Med Ethics. 2014 Jan 10;15:2. doi: 10.1186/1472-6939-15-2 (PMC3897892; doi:10.1186/1472-6939-15-2)
Supplement: Additional file 1 — Study Questionnaire: An English translation of the questionnaire and instructions given to participants. [file 1472-6939-15-2-S1.doc]

**Part I**

**The following ten statements represent potential purposes of the informed consent process. Please rank them from 1 to 10. Rank 1 should be assigned to the statement that you think most reflect the purpose of the informed consent process as it is currently practiced at King Faisal Specialist Hospital & Research Center. Rank 10 should be assigned to the statement that you think least reflect the purpose of the informed consent process as it is currently practiced at King Faisal Specialist Hospital & Research Center. Use each rank from 1 to 10 only once. The statements are presented in a random order.**

**_______ To protect the hospital and its employees from litigations**

**_______ To take away patients’ rights of compensation for injuries that are mentioned in the Informed Consent document**

**_______ To help the patient make his/her own rational medical decision**

**_______ To document patient’s decision**

**_______ A meaningless routine paper work**

**_______ To have a shared decision between patient and clinician**

**_______ To inform the patient**

**_______ To find out what the patient values and prefers**

**_______ To make sure that the patient understands**

**_______ A courtesy gesture**

**Part II**

**The following ten statements represent potential purposes of the informed consent process. Please rank them from 1 to 10. Rank 1 should be assigned to the statement that you think most reflect the purpose of the informed consent process as it should be practiced. Rank 10 should be assigned to the statement that you think least reflect the purpose of the informed consent process as it should be practiced. Use each rank from 1 to 10 only once. The statements are presented in a random order.**

**_______ A courtesy gesture**

**_______ To help the patient makes his/her own rational medical decision**

**_______ To take away patients’ rights of compensation for injuries that are mentioned in the Informed Consent document**

**_______ A meaningless routine paper work**

**_______ To document the patient’s decision**

**_______ To inform the patient**

**_______ To make sure that the patient understands**

**_______ To protect the hospital and its employees from litigations**

**_______ To find out what the patient values and prefers**

**_______ To have a shared decision between patient and clinician**
